# Balram Bhargava: catalysing innovation

**DOI:** 10.2471/BLT.19.031119

**Published:** 2019-11-01

## Abstract

Balram Bhargava talks with Sophie Cousins about fostering affordable, need-driven innovation.

SC: *What inspired you to become a cardiologist?*

BB: Well, first of all, medicine runs in my family. My father was a physician and a pharmacologist. But the event that really set me on the road I have followed was my father having a heart attack, which he fortunately survived, when I was 14 years old.

SC: *You are professor of cardiology at the All India Institute of Medical Sciences (AIIMS), Director General of the Indian Council of Medical Research, Executive Director of the School of International Biodesign, and Secretary at the Department of Health Research in the Ministry of Health and Family Welfare. How do you find time for it all?*

BB: [Laughs]. I am lucky to have the full support of my family! My wife is supportive of everything I do. Also, I consider what I do to be important, unfinished, urgent work. That conviction drives me to make time.

SC: *With all your different responsibilities, do you still find time to see patients?*

BB: I continue to run cardiology and hypertension clinics and see patients there once a week.

SC: *What drew you to biomedical innovation?*

BB: I started taking an interest in biomedical innovation when I was working with Ulrich Sigwart at the Royal Brompton Hospital in London in 1995. Sigwart is credited with pioneering the clinical use of self-expanding vascular stents and I was exposed to some of the innovative aspects of his work there. I also gained insights into why medical devices are so expensive and realised that in India we needed to develop some lower-cost alternatives if we were to ensure access to treatment. It was with that aim in mind that I started working on the development of a low-cost platinum-iridium coil coronary stent.

SC: *Platinum-iridium doesn’t sound particularly low-cost. *

BB: The stent was a good deal cheaper than the products already on the market. The platinum was needed to reduce arterial narrowing which is a major problem within stented arteries and the iridium was added for strength. My brother-in-law is a jeweller in Mumbai and he made the alloys. The metal was later shaped into a fish-scale design and the stents were tested in animals. They were found to be comparable to the best stents in use at that time. Unfortunately, by the time the stent was ready for market, the design was out of date. 

That was an important lesson for me. I realized that we needed to establish an entrepreneurial ecosystem to encourage faster product development. It was at about that time that I met Paul Yock, who headed the Stanford Biodesign Programme and the idea of the Stanford-India Biodesign programme started to take shape. A few years later I co-founded the British Medical Journal publication, BMJ Innovations.

SC: *I understand Stanford-India Biodesign was a collaboration between Stanford University, the All India Institute of Medical Sciences and the Indian Institute of Technology. Is that correct?*

BB: Yes, with funding from the government’s Department of Biotechnology.

“We want to catalyse the development of the Indian medical technology industry.”

SC: *How did the collaboration work?*

BB: The initial idea was to send students for training at Stanford for six months. They then returned to Delhi to identify clinical needs and generate context-specific solutions and business models. The original concept was very focused on robotics and other high-end technologies. It has since become more grounded in local Indian realities and constraints. 

These days the programme works with a range of partners including Japan’s Hiroshima University and Tottori University, the Queensland University of Technology, and, of course, Stanford. The programme aims to train the next generation of medical technology innovators in India and to support the commercialization of novel medical technologies designed for the Indian context. Basically, we want to catalyse the development of the Indian medical technology industry.

SC: *How do you do approach that task?*

BB: We start by carefully selecting a group of young entrepreneurs. Intake is limited to just eight Indian and four foreign students who enter the one-year fellowship programme. In the first quarter students visit clinics and tertiary health care providers, particularly in rural areas, to identify challenges and problems. They then develop products based on their observations. If they are successful, they file a patent and set up a company. Since the programme started in 2007 we have been able to create 14 start-ups developing more than 15 medical devices.

SC: *Can you give some examples?*

BB: The devices include a low-cost, non-invasive device that screens newborns for hearing impairments, designed for mass screening of neonates in resource-constrained settings. The device is being piloted in different Indian states and has also been exported to Uganda and Tanzania. Other devices include a faecal incontinence management device that has been approved by the Food and Drug Administration in the United States of America and a lightweight disposable cast for fractures.

SC: *What do you consider the main challenges to biomedical innovation in India today?*

BB: A big challenge is keeping the focus on what is needed rather than what is profitable or cool. One of the dangers with technological advances is that innovations are introduced for the sake of innovation or because they are perceived as the ‘next big thing’ and, of course, because there is money to be made. This is true for example of artificial intelligence (AI). 

Many physicians in India – and elsewhere – are enamoured of the AI industry and believe it to be the solution to everything. While I believe there are suitable applications for AI, I think we must not let it get between clinicians and their patients. The breakdown of the clinician patient relationship is already happening because of what I call Doctor Google, the marketing efforts of the pharmaceutical industry, and the so-called ‘defensive medicine’ arising as a result of litigation, often driven by opportunistic lawyers. All these things are threatening the doctor-patient relationship. 

One symptom of this breakdown is overdiagnosis and overtreatment, which is being driven by different stakeholders seeking to maximize profits. In India, a lot of sophisticated tests are available over-the-counter, often supported by marketing campaigns and this leads to over-treatment. Magnetic resonance imaging is one example.

“We must not let (Artificial Intelligence) get between clinicians and their patients.”

SC: *How concerned are you about ‘over-diagnosis’ and ‘over-treatment’?*

BB: The amount of investigative medicine that is happening worries me a lot. Stakeholders including hospitals, diagnostic laboratories, non-governmental organizations, and drug makers are all promoting testing through mobile phone messages, media campaigns and flyers. The increase in investigative medicine at the expense of traditional clinical practice is not only increasing the cost of health care, it is breaking the trust between doctor and patient. I started the Society for Less Investigative Medicine (SLIM) at AIIMS precisely to address this issue. We are currently developing standard treatment guidelines for 100 diseases which take account of what is available and what is cost-effective for India.

SC: *Have you had much pushback from the medical community?*

BB: Yes, we have had pushback from many physicians. There are lots of ongoing debates and discussions.

SC: *India just celebrated the first anniversary of the Ayushman Bharat health insurance programme. What is your assessment of the progress made so far?*

BB: I think we are trying very hard to bring health care to the people and the whole world is watching. The intent and commitment are there, but the programme is still in the development phase. The fact is we should not envisage providing unnecessary and expensive treatments for everyone in India, so we need to be very pragmatic and try to cut down on overspending and provide evidence-based rational care. 

As part of Ayushman Bharat, high-end cost-effective secondary and tertiary care is being made available for nearly 100 million families, and 150 000 Health and Wellness Centres are being set up in villages across the country for preventive and promotive healthcare. It’s a massive undertaking, and a huge challenge to focus on health promotion and preventive health when we’re battling cancer, stroke, mental health – health issues that are still emerging. We’re having to come up with new ways to ensure health coverage for those in need.

SC: *Can you give examples?*

BB: Well, some of the examples are quite well known, such as community health workers using smart phones to deliver and monitor different health services. Closer to home, and closer to my heart, so to speak, is the DELHI initiative (Delhi Emergency Life Heart-attack Initiative) which is being piloted by the Indian Council for Medical Research in Delhi and which uses paramedics to get emergency care to patients using motorcycles.

SC: *How does it work?*

BB: People living within 3 kilometres of AIIMS can call a number and within 10 minutes a paramedic and a nurse will arrive at their home. If indicated, the health worker administers thrombolytic therapy through an intravenous line under remote medical supervision and then waits for the ambulance to arrive. According to international data, the outcomes are almost as good as doing angioplasty, if treatment is carried out very rapidly. In a country the size of India we cannot hope to set up cardiac catheterization facilities in every remote village. I hope to scale up the programme across the country soon.

SC: *So, innovation doesn’t always have to be cutting edge.*

BB: That’s right. Effective innovation is innovation that achieves ‘more for less for more’. That’s the motto of the School of International Biodesign, which has a mandate to promote Global Affordable Need Driven Healthcare Innovation, the acronym for which is GANDHI.

